# Neurological sequelae of COVID-19: a review

**DOI:** 10.1186/s41983-021-00379-0

**Published:** 2021-09-08

**Authors:** Christopher J. Peterson, Ashish Sarangi, Fariha Bangash

**Affiliations:** 1 Menninger Department of Psychiatry, Baylor College of Medicine, One Baylor Plaza, Houston, TX 77030 USA; 2 Department of Psychiatry, Texas Tech University Health Sciences Center, 3601 4th St., Lubbock, TX 79430 USA; 3 SUNY Upstate Medical University, 750E Adams St, Syracuse, NY 13210 USA

**Keywords:** COVID-19, SARS-CoV-2, Neurological, Long term, Sequelae

## Abstract

**Background:**

The COVID-19 pandemic has produced a myriad of challenges, including identifying and treating neurological sequelae.

**Main body:**

COVID-19 can cause olfactory and respiratory dysfunction with average recovery within 1 month and a minority of patients experiencing symptoms at 8-month follow-up. Headaches are also very common (up to 93%) amongst patients with persistent COVID-19 symptoms. COVID-19 illness may also affect cognition, although results are mixed.

**Conclusion:**

While many studies have focused on acute COVID-19 symptoms, more longitudinal studies will need to assess the neurological sequelae of the disease. Furthermore, care must be taken when attributing sequelae to COVID-19 illness and not an unrelated cause. Finally, there is concern that COVID-19 may be associated with secondary illnesses, such as Guillain–Barre, and may even contribute to the development of diseases, such as Alzheimer’s.

## Background

The coronavirus disease 2019 (COVID-19) pandemic has presented many challenges, not the least of which is understanding the sequelae effects of the disease. While the early phases of the pandemic understandably focused on the acute clinical presentation of illness, as infection rates begin to fall globally it will be crucial to understand the lasting effects of COVID-19 illness. Neurological sequelae are of special interest both because of their potential to be life-altering as well as the seemingly unexpected nature of these effects for a respiratory virus. While a tremendous effort has been undertaken to prevent and treat severe acute respiratory syndrome coronavirus 2 (SARS-CoV-2) infections, physicians will also be tasked with identifying and treating lingering effects of SARS-CoV-2 long after patients have cleared the virus.

Possible acute neurological consequences of COVID-19 range from olfactory and gustatory dysfunction to encephalopathy and stroke [1]. Up to 90% of COVID-19 patients have reported to have at least one neurological symptom [2]. Even patients without observable neurological manifestations exhibited neurological microstructure changes at 3-month follow-up [3], suggesting that neurological effects could be much more widespread than anticipated.

The etiology for COVID-19 neurological dysfunction is still under investigation and the proposed causes are multi-faceted. The proposed etiologies are based on data from other coronaviruses as well as clinical observations, animal studies, and radiographic imaging. For example, SARS-CoV, as well as Middle Eastern Respiratory Syndrome (MERS) virus, have both been shown to cause neurological symptoms [4] and have also been observed in brain and CSF tissue [5]. Similarly, SARS-CoV-2 has been identified in central nervous system (CNS) tissue including the brainstem [6]. Inflammation, possibly as a result of a cytokine storm, may also contribute to neurological dysfunction [7]. This includes “smoldering”, or low-grade inflammation, that does not produce clinical signs but may contribute to long-term dysfunction [8]. Inflammation may also compromise the blood–brain barrier and allow SARS-CoV-2 (or damaging substances) to access the CNS [9]. Infection with SARS-CoV-2 may also initiate protein aggregation via heparin-binding sites and/or spike-derived peptides, leading to amyloid fibrils that may produce similar pathology to Alzheimer’s disease [10]. Elevated levels of amyloid-beta, neurofilament light, neurogranin, and tau in patients 1–3 months after recovery from COVID-19 suggest generation of neurotoxic proteins may contribute as well [11].

Other causes of neurological dysfunction in COVID-19 may be more indirect. For example, cerebral hypoxia and hypoperfusion due to respiratory dysfunction may cause neuronal damage [12, 13]. COVID-19 may also exacerbate existing neurological disorders due to reallocation of resources and limited access to healthcare [14–16]. Intensive care unit (ICU) admission has been associated with long-term neurological effects and, given the high rates of ICU admission for SARS-CoV-2 [17], may also be a contributing cause. Residual pathology may also result from cardiovascular issues during acute illness, such as stroke [4, 18, 19]. Indeed, D-dimer, plasminogen activator inhibitor 1 (PAI-1), and Von Willebrand factor (VWF) are elevated in COVID-19 patients and may indicate hypercoagulable states and/or endothelial damage that can lead to stroke and other vascular complications [20–23]. SARS-CoV-2 may also induce host production of autoantibodies that can cause pathologies, such as Guillan–Barre syndrome [5]. Furthermore, Graham and colleagues found that a higher prevalence of autoimmune diseases and elevated anti-nuclear antibody (ANA) titers in patients with so-called “long COVID”, suggesting an autoimmune component of disease [24].

Given the prevalence and seriousness of neurological illness associated with COVID-19, greater emphasis on the neurological sequelae of COVID-19 is needed. Here we discuss and summarize current observations from the literature regarding COVID-19 and neurological sequelae. We noted that in some cases these effects are correlated with COVID-19 illness and may not necessarily be the result of COVID-19 pathology. Nevertheless, while significantly more research is needed to elucidate the causes and persistent effects of a SARS-CoV-2 infection, we submit that this review provides important groundwork for researchers and clinicians moving forward.

## Main text

### Methods

The following terms and combinations of terms were searched in PubMed (https://pubmed.ncbi.nlm.nih.gov/) database for the following terms: “COVID-19” OR “long COVID” AND “brain”, “chronic”, “CNS”, “cognition”, “dementia”, “Guillain–Barre”, “headache”, “long-term”, “mechanism”, “memory”, “MRI”, “neurological”, “olfactory”, “outcome”, “Parkinson's”, “recovery”, “seizure”, “sequelae”, “stroke”, and “taste”. Filters for case reports, clinical study, observational study, and prospective study were occasionally used to focus results. Letters to the editor, brief commentaries, book chapters, and pre-prints were excluded. Results published or made available after July 15^th^, 2021 were excluded. Strictly psychiatric conditions such as depression and post-traumatic stress syndrome (PTSD) were excluded. As COVID-19 is an emerging disease, strict timeframes for defining neurological sequelae were not used. Consequences or complications were included based on persistence after either discharge, resolution of other COVID-19 symptoms, or designation as “long term” by study authors.

No ethical approval was obtained or required for the purposes of this review.

### Sensory

Olfactory dysfunction (OD) and gustatory dysfunction (GD) are by far the most studied neurological symptoms of COVID-19 and have generated a tremendous number of clinical studies in a short timeframe. This may be due to both the rapidity of producing these studies (such as via patient surveys), the distinct relationship between OD and SARS-CoV-2, and the publicity surrounding COVID-19 and loss of smell. Indeed, loss of smell and taste is a clinical symptom of COVID-19, and loss of smell is considered a prodromal sign [25]. While the mechanism for olfactory dysfunction is unclear, high levels of SARS-CoV-2 have been observed in the nasal cavity [26] and it is proposed that SARS-CoV-2 can access the olfactory nerve via the angiotensin-converting enzyme 2 (ACE2) and transmembrane serine protease 2 (TMPRSS2) receptors on olfactory epithelium [27], with sensory neuron access via CD147 and neuropilin 1 (NRP1) receptors [28]. Damage to the olfactory nerve (or other tissues) may then occur via inflammation and vascular damage [5]. Reported sensations of “nasal burning” could suggest involvement of the trigeminal nerve [29]. ACE receptors have been widely discussed as a possible receptor in COVID-19 pathogenesis and their presence in CNS tissue may explain COVID-19 associated neurological disease [23, 30]. Furthermore, lower sensory impairment in children with COVID-19 may correlate with age-related expression of ACE2 [31]. Higher rates of OD have been observed in the United States and European populations compared to Asian populations which, given that differences in ACE expression have also been observed between these groups, supports the involvement of ACE2 receptors in OD [32, 33]. However, conflicting data regarding SARS-CoV-2 neuroinvasion exist [34] and some studies suggest olfactory epithelial damage may be the main cause of OD [35].

Central nervous system (CNS) involvement may also play a role in SARS-CoV-2 sensory impairment. The virus may gain access to CNS structures via the olfactory nerve [36, 37], blood, or leukocyte migration across the blood–brain barrier [38]. SARS-CoV-2 can invade the olfactory bulb [39–41]. Magnetic resonance imaging (MRI) findings support a CNS etiology, with observations including olfactory cleft opacification [42] and enlarged olfactory structures (such as olfactory cortices and hippocampi) [43, 44] in COIVD-19 infected or recovering patients. However, rapid recovery of patients with OD suggests a non-neuronal cause, such as damage to olfactory epithelium cells [33, 45–47]. While there is significantly less discussion regarding the mechanism of GD, it is proposed to occur via a similar mechanism (through ACE receptors in the tongue and oral cavity)[48] or via an increase in bradykinin levels [49].

Concomitant olfactory obstruction and rhinorrhea may obfuscate a unique SARS-CoV-2 contribution to OD, although several studies have found no correlation between these symptoms and OD [29, 42, 50–52]. Olfactory dysfunction has been reported with other coronavirus infections and epidemics, including the SARS-CoV virus [53], providing precedence for the OD caused by SARS-CoV-2. What’s more, two studies found COVID-19 patients had a longer duration (10 vs. 15 days, *p* < 0.006) and lower recovery (57.7% vs. 72.1%, *p* = 0.027) than COVID-19 seronegative patients with olfactory dysfunction, suggesting that OD in COVID-19 has a unique etiology (although false negatives could complicate these results) [54, 55]. Therefore, while the exact etiology is unclear, it is likely that SARS-CoV-2 produces sensory impairment irrespective of other upper respiratory symptoms.

Sex and age differences have been reported, with some studies observing a higher incidence (*p* < 0.011) [50] and longer duration (26 vs. 14 days, *p* = 0.009) of OD in females than males, including higher persistence at 4–6-week follow-up (OR 2.46, 95% CI 1.46–4.13, *p* = 0.001) [49, 55]. Conversely, Amer and colleagues noted that females had better recovery at 1 month from COVID-19 onset (83.9% vs. 62.5%, *p* < 0.005), proposing elevated androgens in males that enhance transcription of TMPRSS2 [56]. However, other studies have found no association between sex and OD/GD, making the association unclear [57–59]. Increased age (> 55 years) has also been associated with longer duration of GD (21.6 vs. 33.61 days, *p* = 0.019) [60], whereas ages > 60 years have been correlated with longer durations of OD (*p* < 0.001) [61].

Patients with sensory impairment have varying recovery times. OD symptoms typically resolve by 1–2-month follow-up [58, 59, 62–66], although a range of recovery times have been reported, with some studies reporting a majority of patients recovering as early as approx. 2 weeks [67, 68] and a minority of patients experiencing OD at 7-month follow-up [69]. Of interest are the minority of patients whose symptoms persist after several weeks [70, 71]. For example, Riestra-Ayora and colleagues noted persistence of OD in 11.2% (14/125) patients at 6-month follow-up [72] and Nguyen and colleagues noted 24.0% (30/125) 7 months after onset [69]. Timeframe for recovery from GD is similar to that for OD [62], although GD may resolve more rapidly in some instances [73]. In some rare cases (3/151), OD and GD symptoms have been found to reoccur (mean 19 days from initial resolution of symptoms) [74]. Parosmia has also been noted to occur later in the disease course, with Hopkins et al. showing a median onset of 2.5 months after onset of OD [75]. Indicators of sensory outcome have been observed, with increased severity of illness and persistence of symptoms OD and GD symptoms beyond 10 and 20 days, respectively, correlating with persistent dysfunctional illness [59, 76]. However, Cho and colleagues found no correlation between recovery time based on viral loaded determined by polymerase chain reaction (PCR) [77].

Follow-up for OD and GD studies is usually performed using a questionnaire or using an olfactory/gustatory battery. While these studies are useful, they are nonetheless limited by the range and frequency of follow-up, participant subjectivity, interference of anosmia with gustatory function, and the subjectivity of self-rating systems, as well as an observed discrepancy between objective and self-reporting testing [78–80]. For example, it may have OD on objective testing but fail to perceive such on subjective testing, suggesting that studies that rely solely on self-reporting may underestimate recovery times [80] and vice versa [29].

Several treatments for olfactory dysfunction, typically involving a nasal corticosteroid have been performed with mixed results. For example, one study found betamethasone drops were not significant compared to placebo [81] but found to be significant when combined with ambroxol and rinazine [46]. Groups of patients in these trials are typically small, which may account for conflicting results between studies. Other treatments have shown potential, including fluticasone [82], although others, such as mometasone furate, oral steroids, and nasal irrigation have not [63, 83]. The Clinical Olfactory Working Group currently supports olfactory training as a treatment, although notes that future research is needed for other therapies [84].

### Headache

Headache is a non-specific clinical symptom that has been found to persist after acute COVID-19 infection and may occur in up to 63% of COVID-19 patients [85, 86]. Headache persistence beyond 30 days varies from 3.6% (5/139) [86] to 37.8% (28/74) [87]. However, among patients with symptoms persisting beyond 28 days (n = 558) and 56 days (n = 189), intermittent headaches were one of the most commonly reported symptoms at 91.2% and 93.7%, respectively [88]. Furthermore, Caronna and colleagues noted that 50% (14/28) of patients with persistent headaches had no prior history of headache. Nevertheless, the mechanisms for headaches are unknown but proposed to include elevated cytokines and chemokines [87, 89], activation of trigeminal nerve branches, and ACE2 interaction in cerebral blood vessels [87]. Pediatric patients, a population not normally considered with COVID-19, may have neurological sequelae, such as headache and difficulties with concentration, although the evidence is limited [90, 91].

### Cognition and memory

Cognition and memory can be chronically affected by COVID-19. Miskowiak and colleagues found an association between cognitive impairments and D-dimer levels during the acute phase of illness [12], supporting previous studies that use of heparin and tissue plasminogen activator (tPA) may improve outcomes [92, 93]. Cognitive recovery varies, with some studies showing significant recovery in the majority of COVID-19 patients at 1 month [94, 95], with Rass and colleagues found that 23% of studied patients had cognitive defects (measured by Montreal Cognitive Assessment) at 3-month follow-up. This was increased to 50% among those who had encephalopathy during COVID-19 illness [79]. McLoughlin et al. found that, among previously hospitalized adult patients with COVID-19, cognition at 4-week follow-up (measured using the modified Telephone Instrument for Cognitive Status) was similar to those without delirium. However, physical function (as measured by combined Barthel Index and Nottingham Extended Activities of Living scores) was markedly worse in those with delirium (97 vs. 153, *p* < 0.01) [96]. Furthermore, higher rates of dementia were observed among COVID-19 survivors compared to those with influenza (HR 2.33; 95% CI 1.77–3.07; *p* < 0.0001) or other respiratory infections (HR 1.71; 95% CI 1.50–1.95) [97]. Interestingly, van der Borst and colleagues, in a study of 124 COVID-19 patients (46 with severe or critical disease), found that mental or cognitive function was not correlated with disease severity via the Clinical Frailty Scale [98]. Conversely, a study of 120 participants with mild–moderate COVID-19 observed that 98.3% (118/120) had normal cognitive functioning at 4-month follow-up. However, this group had a relatively low rate of comorbidities (15% hypertension, 8.3% obesity, 3.3% diabetes) and rarely received supplementary oxygen therapy (1.6%) [99]. Finally, inpatient rehabilitation was associated with significant improvement in memory and cognitive function in a cohort (n = 29) of hospitalized COVID-19 patients (mean hospital and intubation length 32.2 and 18.7 days, respectively), with 90% of patients discharged home after a mean of 16.7 of inpatient rehab [100], suggesting the importance of such care in recovery from COVID-19.

There is also concern that COVID-19 may cause or worsen neurological diseases, such as Alzheimer’s. For example, apolipoprotein E (ApoE) e4e4 allele, highly associated with Alzheimer’s, may increase the risk of severe COVID-19 [101]. Risk for Alzheimer’s might be indirectly increased due to respiratory dysfunction, as tau hyperphosphorylation is increased by hypoxia [102]. Alzheimer’s patients could be more vulnerable to COVID-19 given increased ACE expression in brains of Alzheimer’s patients has been observed [9]; tau hyperphosphorylation increased due to hypoxia.

### Other neurological disorders

Several other neurological conditions have been reported in COVID-19 patients. Cavalagli and colleagues reported cranial nerve V3, IX, X, and XII impairment with gradual recovery over 2 months in a 69-year-old male [103]. Akinetic mutism and transient non-specific frontal release signs have been reported in 13.1% (8/61) of COVID-19 ICU patients at 3-month follow-up [104]. COVID-19 may also negatively affect quality of sleep. Rass and colleagues noted that 34% (n = 135) of patients had sleep disturbances at 3-month follow-up, with higher rates among ICU patients (43%, n = 31). The authors note that fatigue was more frequent in patients with sleep disturbances (adjOR 6.39; 95% CI 2.63–15.53, *p* < 0.001), which may partially explain fatigue observed in “long COVID” patients. Sleep disturbance was also associated with new neurological disease (adjOR 5.71; 95% CI 1.76–18.54; *p* = 0.004) [79]. Huang and colleagues, in a large survey of discharged COVID-19 patients, observed 2% (437/1655) of patients had sleep difficulties at 6-month follow-up, with similar rates for those requiring (26%; 290/1114) and not requiring (27%; 116/424) supplemental oxygen [105].

Stroke and COVID-19 are of concern due to hypercoagulable states induced by COVID-19 infection [106] and possible infection of vasculoendothelial cells [23, 38]. Furthermore, limitations in health resources due to the pandemic, suggested by markedly decreased rates of stroke admission and emergency care early in the pandemic, may also indirectly contribute to long-term effects of stroke in non-COVID-19 patients [107, 108]. Given that stroke can be neurologically devastating, monitoring for stroke and stroke prevention may need to be considered in at-risk COVID-19 patients. COVID-19 may also longitudinally impact patients with Parkinson’s disease, although the evidence is limited. Leta and colleagues observed “post-COVID syndrome” (defined as symptoms persisting beyond 12 weeks) in 27 patients with Parkinson’s, noting worsening motor function (51.9%) and increased levodopa requirements (48.2%) [109]. Finally, there have been a number of Guillain–Barre (GBS) cases associated with COVID-19, with one study identifying over 70 cases [19, 110], although a causal link has not been established. A multi-center Italian study noted a 2.6 fold increased in GBS cases in March and April of 2020 compared to the same period in 2019 and estimated GBS incidence among COVID-19 positive patients (47.9/100,000/year) and COVID-19 positive hospitalized patients (236/100,000/year) [111]. Abu-Rumeileh and colleagues noted a wide age range of affected patients (11–94 years, mean 55 years) with male predominance [110] Furthermore, demyelinating lesions have been observed in the brain and spinal cord on CT and MRI, possibly from an autoimmune etiology similar to GBS or multiple sclerosis [112]. Given that recovery from Guillain–Barre can take months to years [113], the effects of secondary illnesses associated with COVID-19 can be potentially devastating.

## Conclusions

The long-term neurological complications of COVID-19 are varied and still poorly understood. While many studies have assessed the clinical impact of COVID-19 on the nervous system, most of these are limited in scope. Many studies have follow-up times less than 3 months, have limited number of patients (such as case reports or case series), and use surveys instead of clinical or radiological assessments. We also note that many proposed mechanisms of COVID-19 neuropathology are theoretical and not based on empirical evidence. While these initial clinical observations and proposed mechanisms provide useful starting points for future research, they are neither sufficient nor definitive. Long-term prospective studies employing sensory and radiological assessments will be crucial for assessing the impact and progression of long-term COVID-19 neurological symptoms. While the long-term impact of COVID-19 is still undetermined, clinicians should be aware of possible outcomes and etiologies when caring for patients recovering from this virus.

## Data Availability

Data sharing is not applicable to this article as no data sets were generated or analyzed during the current study.

## Abbreviations

COVID-19: Coronavirus disease 2019
SARS-CoV-2: Severe acute respiratory syndrome coronavirus 2
MERS: Middle eastern respiratory syndrome
CNS: Central nervous system
SARS-CoV: Severe acute respiratory syndrome
ICU: Intensive care unit
ANA: Anti-nuclear antibody
MRI: Magnetic resonance imaging
OD: Olfactory dysfunction
GD: Gustatory dysfunction
ACE: Angiotensin-converting enzyme
TMPRSS2: Transmembrane serine protease 2
PCR: Polymerase chain reaction
tPA: Tissue plasminogen activator
ApoE: Apolipoprotein E
GBS: Guillain–Barre syndrome
CT: Computed tomography
MRI: Magnet resonance imaging
adjOR: Adjusted odds ratio
OR: Odds ratio
HR: Hazard ratio
CI: Confidence interval
